# Effect of Surgical Installation of Dental Implants on Surface Topography and Its Influence on Osteoblast Proliferation

**DOI:** 10.1155/2018/4089274

**Published:** 2018-10-17

**Authors:** Helder H. M. Menezes, Marina M. Naves, Henara L. Costa, Tarsis P. Barbosa, Jéssica A. Ferreira, Denildo Magalhães, Elizabeth F. Martinez

**Affiliations:** ^1^Department of Periodontology and Implant Dentistry, HD Post Graduation Dental School, Uberlândia 38408-394, Brazil; ^2^School of Dentistry, Federal University of Uberlandia, Uberlândia 38405-320, Brazil; ^3^School of Mechanical Engineering, Federal University of Uberlandia, Uberlandia 38400-901, Brazil; ^4^School of Engineering, Federal University of Rio Grande, Rio Grande 96203-900, Brazil; ^5^Department of Telecommunications and Mechatronics, Federal University of Sao Joao Del-Rei, Ouro Branco 36420-000, Brazil; ^6^Department of Cellular and Mollecular Biology, São Leopoldo Mandic Research, Campinas 13045-755, Brazil

## Abstract

Surface treatment alone does not determine the final microtopography of a dental implant, which can be influenced by implant design and the surgical procedure. This study investigated the effect of surgical placement of dental implants with same surface treatments on surface roughness. Three implants (SIN) of each group with different macrogeometries (Strong, Stylus, and Tryon) were analyzed using laser interferometry and scanning electron microscopy to evaluate surface topography. All threaded regions of the implants, namely, top, flank, and valley, were analyzed individually. Relevant surface parameters (*S*_a_, *S*_sk_, *S*_ku_, *S*_tr_, and *S*_dq_) were calculated for the different regions on each implant before (B) (*n* = 9) and after (A) (*n* = 9) placement into porcine rib bones. The behavior and proliferation of a preosteoblastic cell line MC3T3-E1 on titanium surface, cell viability, and osteopontin secretion were evaluated after 24 h, 48 h, and 96 h, also before (*n* = 18) and after (*n* = 18) implant placement into porcine ribs bone. As results, the valleys of all implants had an increase in *S*_a_ values after implant placement. By contrast, the tops of the Stylus A implant and the flanks of the Tryon A implant showed a significant decrease in mean height of the irregularities (*S*_a_), 0.16 *µ*m and 1.25 *µ*m, respectively. The Stylus implant presented significantly (*p* < 0.05) higher asymmetry values on the distribution curve for irregularity heights (*S*_ku_) in all regions after insertion into bone (6.99 for tops, 9.54 for flanks, and 17.64 for valleys), indicating a greater preponderance of peaks over valleys. An increase in roughness gradients (*S*_dq_) was observed for all macrogeometries after insertion into bone. The cell culture results showed no significant difference (*p* > 0.05) for all macrogeometries after bone placement. In conclusion, a subtle change in implant surface roughness was detected after insertion into bone for all the macrogeometries, without significantly affecting the cellular parameters studied.

## 1. Introduction

Long-term success of dental implants is based on several factors [1–3] including osseointegration [4–6]. In addition, the chemical biocompatibility of the material and the microgeometry of the implant surface are important for implant success and long-term osseointegration, affecting the cellular response, resulting in greater/better quality bone formation and, therefore, improving secondary implant stability [7–12].

In order to increase long-term implant success/survival rates, research has focused on implant modifications, including surface topography and macrogeometry [13]. The design of most recent implants seeks to minimize potential osseointegration issues, such as low-density bone or patients with systemic diseases that compromise bone repair, and also to reduce bone trauma and prosthetic fractures, especially when immediate loading is applied [13]. With modification of any of the mechanical or chemical properties of an implant, host tissue response is expected to improve, thus optimizing the bond with the bone tissue [5, 14].

Furthermore, implant morphology can also influence bone metabolism: rougher surfaces stimulate differentiation, growth, and attachment of bone cells and increase mineralization. Implants may have “smooth” (machined) or rough surfaces. The main methods that are reported in the literature to create implant roughness are titanium plasma spraying, hydroxyapatite (HA) coating, sandblasting, and acid etching. A current tendency is the manufacturing of implants with submicro (nano) topography and microtopography. Furthermore, the biofunctionalization of implant surfaces, by adding different substances to improve its biological characteristics, has also been recently investigated [15–17].

The development of bone-implant interfaces depends on the direct interactions of bone matrix and osteoblasts with the biomaterial [18]. Osteoblast adhesion is essential for bone-biomaterial interactions [19]. Consequently, maximization of bone integration has become a goal of the treatment, which apparently can be improved by varying surface roughness of the implant [20]. Therefore, cell adhesion is a fundamental process directly involved in cell growth, cell migration, and cell differentiation. It is concerned in embryogenesis, maintenance of tissue integrity, wound healing, immune response, and biomaterial tissue integration. Several proteins are involved in cell adhesion, such as extracellular matrix proteins (collagen, fibronectin, and vitronectin) and membrane receptors (integrins). Interactions between these proteins and their specific receptors induce signal transduction and consequently influence cell growth and differentiation.

Studies have shown that implants with roughened surfaces have better bone apposition and higher BIC when compared with smooth surfaces [2, 21]. In 2004, Albrektsson and Wennerberg [2] described the topographical properties by a literature review, and the findings showed that moderate roughened surfaces (*S*_a_ between 1.0 and 2.0 *µ*m) present better osseous response than rougher or smoother surfaces.

Changes in implant macrogeometry also contribute to implant success by directly affecting primary stability [22–25]. First, for tapered implants, the macrogeometry of the implant body and the thread design are directly related to the surface contact area of the implant. The area of surface contact with the host tissue will determine contact pressures, affecting implant stability [26–28]. Second, the insertion torque of tapered implants is normally greater than that of cylindrical implants [26]. Among tapered implants, the insertion torque is affected by the thread design (shape, width, depth, pitch, face, and helix angle) and thread pitch (angle and width). The insertion torque will affect the amount of deformation in the bone, changing the amount of bone around the implant and the degree of bone apposition, which will influence bone remodeling, affecting implant success [29–32]. Third, the thread design will affect micromovements of the implant in relation to the bone since it will affect mechanical interlocking.

In screw-type implants, it is necessary to measure topography in three regions of the threads: flank, top, and valley [32–35]. Although this characterization is well defined, it is unclear whether increasingly complex surface features found in modern dental implants are maintained after bone placement.

During implant placement, the insertion torque may result in varying levels of compression stress transmitted to the adjacent bone, since the diameter of the osteotomy is somewhat narrower than that of the diameter of the implant to optimize primary stability [35, 36]. Clinical studies have demonstrated a close relationship between initial stability and implant success [37–40], where the former can be measured by insertion torque during implant placement [38]. Insertion torque, in most cases, should be higher than 30 N cm for predictable outcomes [38], thus preventing implant micromovement and, consequently, connective tissue formation [39]. An excessively high insertion torque above 50 N cm [36] may occur during implant installation in dense bones [37, 40], resulting in compression stress to the adjacent bone and compromising osseointegration [41]. In addition, some studies have shown that shear force during placement may alter implant surface features [42–44].

Considering the lack of studies investigating the stability of surface characteristics after implant placement as well as a lack of clarity in the methods reported to analyze implant design in such context, the present study investigated *ex vivo* the influence of different implant macrogeometry and insertion torque to cortical and cancellous bone in the surface microtopography and cellular parameters *in vitro*, considering cellular proliferation and viability assays, as well as, osteopontin expression.

## 2. Materials and Methods

### 2.1. Sample Description

**Figure 1:**
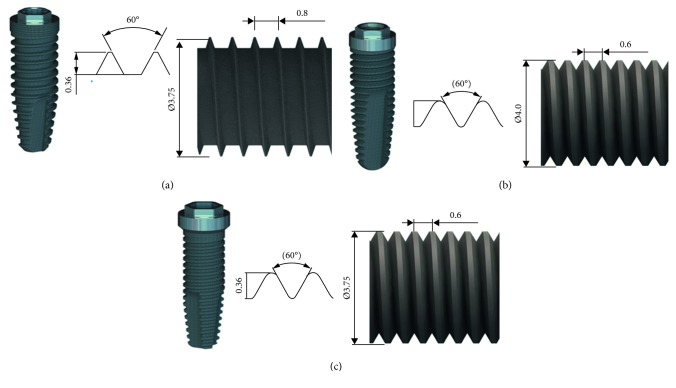
Macrogeometry of the implants and detail of their diameter and threads. (a) Sample of group Strong—implant with smaller thread heights/triangular V shaped (size: 3.75 mm × 13 mm). (b) Sample of Stylus—implant with lower internal thread angles/square and buttress thread-l (size: 4.0 mm × 13 mm). (c) Sample of Tryon—implant with lower internal thread angles/square and buttress thread-(size: 3.75 mm × 13 mm); all implants are hybrid (cervical part of the implant body in cylindrical shape and apical part of the implant body in tapered shape).

This study analyzed commercially pure titanium implants (grade 4) of the types Strong, Stylus, and Tryon, marketed by SIN (Implant System, São Paulo, Brazil). All implants presented an external hexagon connection with a 4.1 mm platform. The Strong and Tryon implants had a diameter of 3.75 mm, and the Stylus implants had a diameter of 4.0 mm, all of which were 13 mm long with different macrogeometries, as shown in Figure 1. For the topographic characterization of the surfaces, three samples from each type of implant were analyzed before (B) and three after (A) installation into 3 porcine rib bones. For the *in vitro* cellular analysis, six samples from each type of implant were analyzed before and after installation into 3 porcine bones.

All samples with the same type of surface treatment were purchased directly from the manufacturer. The treatment consisted of nitric acid followed by sulfuric acid (DAA, double acid etching) baths, according to the description by the manufacturer.

### 2.2. Implant Placement into Porcine Ribs

Nine fresh porcine ribs (bone quality D3) [45] with 15 mm high and 15 cm long were used as an experimental model. Three implants of each macrogeometry were placed into 3 porcine ribs for the implant surfaces analysis (*n* = 9), and six implants of each macrogeometry (*n* = 18) were placed into three ribs for cellular assays analysis. Considering that the animals were not sacrificed for research purposes, this study was exempt from approval by the Animal Ethics Committee [46, 47].

**Figure 2:**
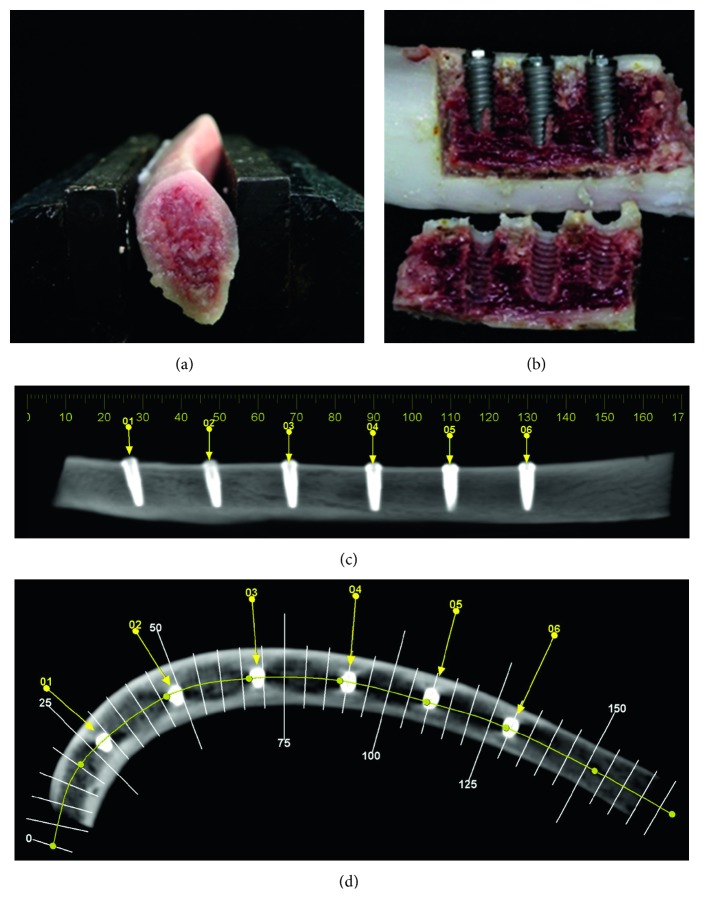
(a) Porcine rib positioned on the vise for implant installation. (b) Vertically sectioned porcine rib with Strong implants after osteotomy with piezoelectric ultrasound. (c, d) Transverse CT of implants into porcine rib.

Dental implants were placed into 13 mm deep perforations, following the manufacturer's instructions (Figures 2(a) and 2(b)), by one expert surgeon, and final drill with dimension of 3.0 mm. The distance between the implants was 20 mm. Implant fixation was measured by the same manufacturer using a manual torque wrench, with a maximum torque of 60 N cm, as recommended by the manufacturer. Computed tomography (CT) was performed to ascertain the exact position of each implant (Figures 2(c) and 2(d)). After installation, the implants were removed using a piezoelectric ultrasonic tip (PiezoSurgery® White, Mectron, Italy) cutting the bone laterally to the implant, taking care not to damage the implant surface and allow passive release of the implants from the bone.

Implants were cleaned to remove bone debris from the surface by immersion in filtered water (30 minutes) and then in acetone (10 minutes) as suggested by Senna et al. [44].

### 2.3. Characterization of Implant Surfaces

Laser interferometry and scanning electron microscopy (SEM) were used to characterize the surface of the implants. A 3D laser interferometer (UBM MESSTECHNIK MicroFocus, Ettlingen, Germany) was used to analyze surface topography at densities of 1000 × 1000 points and measurement rate of 300 points/s, using the continuous method. The measurement area was 0.8 × 0.4 mm to allow the inclusion of at least one thread for all regions without losing focus [48].

**Figure 3:**
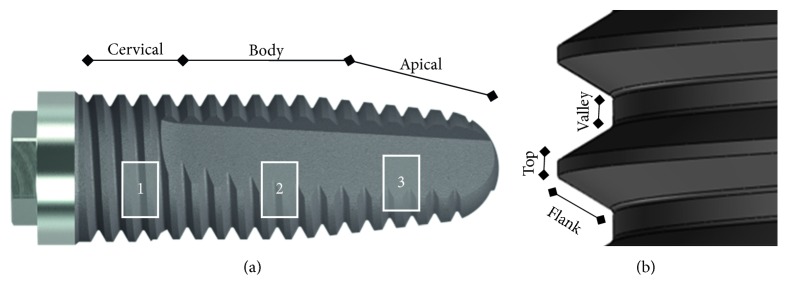
(a) Implant macroregions for analysis: 1-cervical, 2-body, and 3-apical. (b) Analyzed areas of each thread (top, flank, and valley) of regions 1, 2, and 3.

Due to the complex macrogeometry of the implants, different areas were measured for each implant, as shown in Figure 3. From all the samples evaluated (*n* = 18), 9 were measured before installation and 9 after, from which 6 were model Strong, 6 Stylus, and 6 Tryon. Each implant was divided into three macroregions for analysis: 4 mm within the cervical region [1], 5 mm within the implant body [2], and 4 mm at the apical end [3] (Figure 3(a)). Thus, for each region, three tops, three valleys, and three consecutively threaded flanks (Figure 3(b)) measuring 0.8 × 0.4 mm were analyzed and measured, totaling 27 measurements for each implant in each macroregion. All the implants were randomized to minimize the effects of subjective bias.

The characterization of surface topography consisted of three components: shape, waviness, and roughness, where filters were necessary to isolate each of these components for analysis. The software Mountains Map (Digital Surf, Besançon, France) was used for this purpose, which also permitted collation of the 2D and 3D images based on numerical description of the parameters for surface roughness. To characterize the shape of the implants (macrogeometry), profiles of the threads were selected. Vertical heights and thread angles were measured in the profiles. The MB Ruler software (MarkusBader MB-Software solutions, Iffezheim, Germany) was used to calculate the angles of the implant threads, the total length of the implant thread profile, and the specific lengths of every flanks, tops, and valleys [47]. Dividing the total length by the specific lengths of each region, the percentage of the area of each region in relation to the whole surface of the implant was estimated. For the calculation of the roughness parameters, the first step was to use a filter to remove the shape of the implant. Then, a 50 × 50 *μ*m Gaussian filter was used to separate waviness from roughness.

The numerical description of surface roughness at the different regions used three height parameters (*S*_a_, *S*_sk_, and *S*_ku_), one spatial parameter (*S*_tr_), and one hybrid parameter (*S*_dq_). *S*_a_, also referred to as mean roughness, is the arithmetic mean height of the asperities. *S*_sk_ and *S*_ku_ are related to the height distribution curve, where *S*_sk_ is a measure of asymmetry of surface deviations about the mean plane, and *S*_ku_ is a measure of the peakedness or sharpness of the surface height distribution. *S*_tr_ is the texture aspect ratio of the surface, used to identify uniformity of texture aspect. *S*_dq_ is the root mean square slope of the asperities. The mathematical description of these parameters can be found in the study by Stout et al. [49]. Other studies in the literature [31, 48] have used the same parameters to characterize the surface topography of dental implants.

Ultrastructural SEM images of the tops, flanks, and valleys were performed before and after implant installation to provide a qualitative analysis of the surfaces (EVO MA 10, Carl Zeiss, Germany) at different magnifications and 15 kV voltage acceleration. This surface characterization was performed in three implants of each macrogeometry before installation in the porcine rib (B implants) and in three implants after installation in the porcine rib (A implants).

Osteoblast cell proliferation and viability assays, as well as osteopontin quantification were performed on the different surfaces, before and after implants installation. All the assays were performed in triplicates at a density of 1.9×10^4^/well on 24-well tissue culture plates. In order to stabilize the implants inside the 24-well tissue culture plate, orthodontic wire segments were used (nickel-titanium (NiTi) (Morelli Ortodontia™--Sorocaba, SP, Brazil).

The preosteoblastic cell line MC3T3-E1 was obtained from the ATCC (American Type Culture Collection, ATCC, VC, USA) and cultured at 4^th^ to 6^th^ passage in DMEM/F-12 medium (LGC Biotechnology, São Paulo, SP, Brazil), supplemented with 10% bovine fetal serum (LGC Biotechnology) and 100 U/ml penicillin and 100 *μ*g/ml streptomycin (Sigma, St. Louis, Missouri, USA). During the culture period, cells were incubated at 37°C in a humidified atmosphere of 5% CO_2_ and 95% air, and the medium was changed every 2-3 days [48].

For the evaluation of cell proliferation, the trypan blue vital exclusion method was used at 24 h, 48 h, and 96 h from cell seeding onto the surfaces. The cells were enzymatically detached from the surfaces with 1 mM EDTA (Gibco) and 0.25% trypsin solution (Gibco). The cells were then counted using a hemocytometer (Hausser Scientific, Horsham, PA). Cell proliferation was expressed as number of cells × 10^4^.

Cell viability was evaluated by 3-[4,5-dimethylthiazol-2-yl]-2,5-diphenyl tetrazolium bromide (MTT; Sigma) assay after 24, 48, and 72 h. Briefly, the cells were incubated with 10% of MTT (5 mg/mL) in culture medium at 37°C for 4 h. The MTT solution was then aspirated from the well, and 200 *µ*L of dimethyl sulfoxide (Sigma) was added to each well. Then, the plates were agitated on a plate shaker for 5 min, and 150 *μ*L of this solution was transferred to a new 96-well plate. The optical density was read at 570–650 nm on the plate reader (Epoch; Bio-Tek, Winooski, VT), and data were expressed as absorbance.

Quantification of secreted osteopontin by the osteoblastic cells cultured on the different surfaces was evaluated by enzyme immunoassay (ELISA). The supernatant was collected and centrifuged at 336 g for 10 min, and the resulting supernatant was collected, aliquoted, and stored at −80°C. The osteopontin quantification was carried out using Mouse Osteopontin kit (R&D Systems, Minneapolis, USA) according to the manufacturer's instructions. The values were expressed as ng/ml.

### 2.4. Statistical Analysis

For each parameter, mean and standard deviation values were calculated for the implants before and after installation. In order to evaluate statistical significance, the topographical parameters (B and A) were analyzed using the paired *t*-test (*α* = 0.05) (SPSS Statistics Base 17.0-IBM, Chicago, USA).

The Shapiro–Wilk test was used to test normality on the data collected, which did not conform to the assumptions of normality. The Wilcoxon test was paired and used to better compare the mesurements between two samples statistically similar, on dependent samples. The morphological aspects before (B) and after (A) implant installation into bone were compared for the Strong, Stylus, and Tryon macrogeometries. The entire study was performed considering a confidence level of 95%. The comparison among implants from different macrogeometries was not performed because the cell sedimentation region within the flanks and valleys were different across the groups.

## 3. Results and Discussion

### 3.1. Surface Characterization

The implant insertion torque did not exceed the maximum values recommended by the manufacturer, and there was considerable variation in insertion torque among the three implants, possibly due to the influence of the macrogeometry on the porcine rib bone, which is D3. The mean insertion torque (N.cm) was 48.3 3(±10.98) for Strong, 33.88 (±15.58) for Stylus, and 33.05 (±13.84) for Tryon. The porcine rib was chosen due to its cortical thickness (2 mm thick) and microscopic bone structure that resembles the human jaw bone.

Table 1 shows the values of the parameters used to characterize the threads in the different regions for each implant. Some variation in angles and heights between regions for the same implant were observed. The Strong implant presented threads with smaller thread heights, while the Stylus implant had lower internal thread angles. It was possible to observe that the flanks corresponded to the larger area for all implants. The tops were the second largest areas in the Stylus and Tryon implants, while the valleys were the second largest areas in the Strong implant.

The mean values ±standard deviation of the surface roughness parameters (*S*_a_, *S*_sk_, *S*_ku_, *S*_tr_, and *S*_dq_) were the means of all regions 1, 2, and 3 for each type of dental implant (B and A) and are shown in Table 2. The values of total Δ are the difference between the mean of all areas (top, flank, and valley) before and after the implant placement for each implant type, shown in Table 2. Considering the evaluation methods, other studies that evaluated surface alterations also used scanning electron microscopy and interferometry [41–43]. As laser interferometry is suggested as an excellent quantification method for screw-type implant topography [31, 51, 52], the same surface roughness (3D) parameters were evaluated before and after installation in the porcine rib.

**Figure 4:**
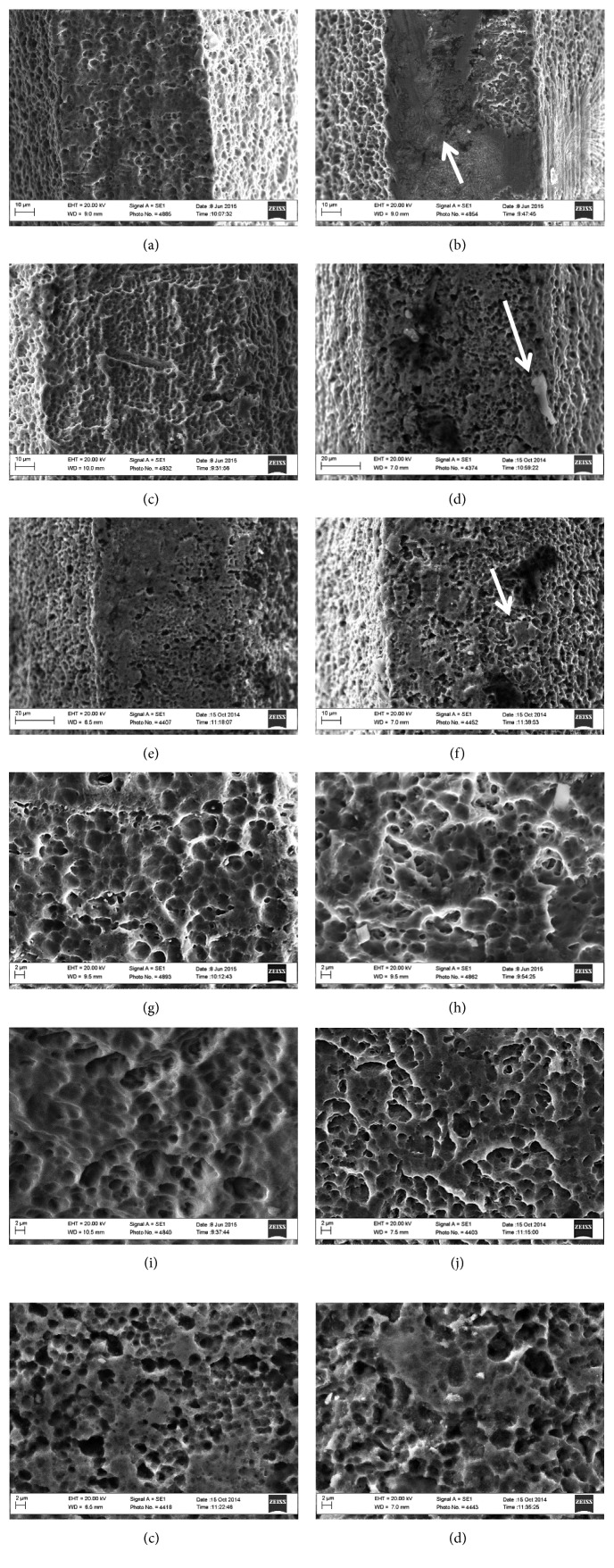
Representative images of the implants prior to placement (a–c) and after removal from the bone (d–f). Although the images are very similar qualitatively in both conditions, it is possible to observe discrete deformation (arrow, (f)) and residue (arrow, (d)) on the implant surface after insertion/removal (A). Surface treatment in some implants appears not effective in covering all regions, such as those indicated by the arrow in Figure 4(b), which has a different surface topography. When the thread tops were seen at higher magnification, their surface morphology was very similar across all implants (g–i). After insertion (A), minimal changes may be noticed, suggesting small plastic deformation in surface irregularities for the three different implants (j–l).

For the tops, flanks, and valleys, implant macrogeometry had a significant influence in *S*_a_ values, mainly for Strong and Tryon implants. Regarding the effects of bone insertion, it was observed that the parameter *S*_a_, which represents the mean height of the surface irregularities, showed significant reduction in the top regions after insertion in the bone for the Stylus (Stylus top *p*=0.000). It must be pointed out that for this implant, the top regions did not present regular topography, i.e., the surface treatment did not seem to have been effective to change the whole area (Figure 4(b)).This seemed to occur as a result of the flattening of the asperities observed from the SEM images, which was much less significant for the top of the implant Tryon. On the contrary, for the Strong implant, *S*_a_ showed a tendency to increase with bone insertion, although the standard deviation was very high. The area of the tops of those implants were smaller compared with the other implants (Table 1), which probably resulted in higher contact pressures during the insertion process. This could lead to more intense generation of bone debris, which may justify their higher values of *S*_a_ and also the larger scattering of the results. Some evidence of bone debris is indicated by the arrow in Figure 4(d) relative to the top of the Strong implant after insertion. Significant reduction in *S*_a_ values on the flanks of the implants was observed for the Tryon implants (Tryon flank *p*=0.000). Regarding the thread valleys, all values of *S*_a_ increased after placement into bone, which was statistically significant in the Strong implants (*p*=0.001) and Tryon (*p*=0.000). This is probably related to the accommodation of bone debris resulting from bone insertion in the valleys of the threads and/or to damage to the valleys of the threads.

According to Albrektsson and Wennerberg [2], implants can be divided into four categories depending on surface roughness: smooth (*S*_a_ < 0.5 *μ*m), minimally rough (*S*_a_ between 0.5 and 1.0 *μ*m), moderately rough (*S*_a_ between 1.0 and 2.0 *μ*m), and rough (*S*_a_ > 2.0 *μ*m). Some studies have suggested that an ideal implant surface should have *S*_a_ values between 1.0 and 2.0 *μ*m [29, 50, 51]. In this study, most implants showed *S*_a_ values below 1.0 *μ*m (minimally rough) prior to insertion into bone (B). One exception was the Tryon implant (B), which presented values of *S*_a_ in the flank of 1.98 ± 0.68 (Table 2). Before insertion into porcine ribs, macrogeometries had little effect on *S*_a_ values, but the insertion process into bone may have led to the formation of bone debris that accommodated in the valleys of the threads and/or simply damaged the thread valleys for the Tryon (A) and Strong (A) implants, increasing *S*_a_ values in these regions. At the tops of the Stylus (A) implant and flanks of the Tryon (A) implant, the *S*_a_ values decreased, apparently due to flattening of the surface irregularities. The fact that roughness parameters were calculated in the different regions (tops, flanks, and valleys) enabled identifying different phenomena occurring during insertion, i.e., flattening of asperities at the flanks and tops of some implants and debris accumulation at the valleys of some implants. Another possible reason for changes in *S*_a_ after the insertion process is the accommodation of smaller bone debris in the valleys of the surface topography, but this should be accompanied by an increase in the values of *S*_sk_, which did not occur, as described below.

The parameters associated with height distribution of surface irregularities are *S*_sk_ and *S*_ku_. The parameter *S*_sk_ is associated with asymmetry of the height distribution curve, where curves with approximately normal distribution present *S*_sk_ values close to 0. *S*_ku_ is associated with the flatness of the height distribution curve, where curves with approximately normal distribution have *S*_ku_ values close to 3. All implant regions of B and A showed *S*_sk_ values close to 0 and *S*_ku_ values close to 3 (Table 2). The *S*_sk_ values for all implants B and A showed no significant changes (Table 2).

Since all regions of all implants showed values close to 0, no top or valley predominance was observed in any region of the implants before or after placement. The deviations of *S*_sk_ from 0 (corresponding to a normal distribution), whether slightly positive or negative, may be considered too small to represent any relevant asymmetry [54]. Although *S*_ku_ values for top and flank increased across all implants after insertion into bone, such findings do not represent physical significance overall, indicating only discrete deformation at the top surfaces. The mean *S*_ku_ values were close to 3 in all regions for all (B) implants. There was a mild increase in these values for the top, flank, and valley of the Stylus (A) implant and to the top of the Tryon (A) implant. Such an increase in *S*_ku_ values may be due to surface roughness modifications during the insertion process [42–44], creating a small number of very sharp peaks. *S*_ku_ values close to 3, presented together with *S*_sk_ values close to 0, confirm that the distribution of irregularity heights is close to a normal distribution, regardless of macrogeometry or implant region.

**Figure 5:**
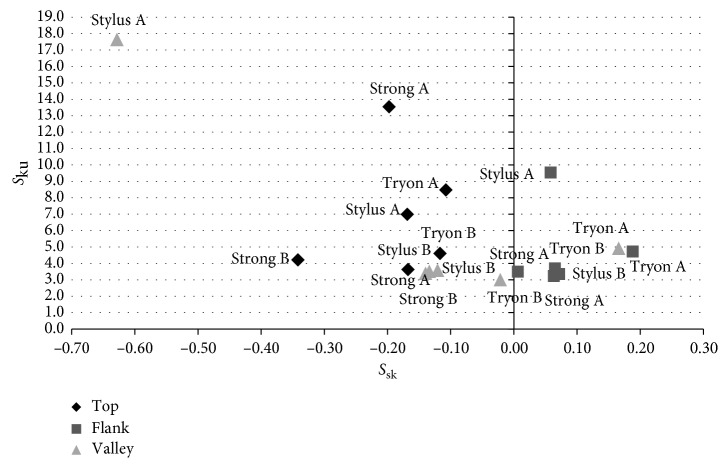
Morphological space of the parameters *S*_sk_(*x*) × *S*_ku_(*y*) for implants B and A.

As these parameters may vary greatly with discrete changes in topography, such as residues, it is prudent to evaluate a plot of morphological space *S*_ku_ × *S*_sk_ before and after bone insertion rather than numbers alone [55].  Figure 5 shows the *S*_ku_ × *S*_sk_ morphological space for all the different regions of each implant analyzed before (B) and after (A) insertion. For all the tops, shown in the plot with the symbols “∗,” *S*_ku_ values increased. The flattening effect due to possible plastic deformation observed for the tops of the implants results that the vast majority of the asperities have similar heights, with the presence of very few sharp peaks, which increases *S*_ku_. For the flanks of the Stylus implant, *S*_ku_ also increased after bone insertion, but no evident flattening mechanism was detected from the SEM images. This increase might be simply due the presence of a few residues such as bone debris, so that, in comparison with the large debris, the heights of the other asperities seem “flattened.”

To quantify the texture strength, i.e., the uniformity of surface texture, the parameter *S*_tr_ was used, which evaluates the texture aspect ratio of the surfaces. Values of *S*_tr_ > 0.5 indicate a uniform texture in all directions, i.e., the surface is topographically isotropic; whereas *S*_tr_ < 0.3 indicates strong directionality of the texture (anisotropy) [49]. The results for implants B and A are shown in Table 2. Initially, it was observed that all implants presented *S*_tr_ < 0.3, i.e., strong anisotropy. All implants B and A presented lower *S*_tr_ values for the flanks and valleys compared with the tops, suggesting a more evident directionality in these regions. After insertion into bone, all implants maintained their anisotropic surface characteristics in all regions. The flanks and valleys of the Stylus and Tryon implants increased their directionality after insertion into bone, presenting a significant reduction in *S*_tr_ values (Stylus flank, *p*=0.000 and *p*=0.000; Tryon flank, *p*=0.008 and *p*=0.000). It was verified that the same surface treatment in different implants resulted in differences in roughness parameters, due probably to variables relating to the double-etching process itself, particularly depending on the macrogeometry of the implant and the thread region within each implant. In general, implants B and A maintained their anisotropic characteristics. Moreover, as this study did not apply reverse torque for implant removal, as suggested by Mint [42] and Senna [44], the results presented herein are more reliable when compared with the Salerno study [43], where values of topography deformation did not show any significant change, due probably to the process of removal, which was based on reverse torque.

The *S*_dq_ values, which represent the mean square values of the roughness slopes, are also shown in Table 2. In general, the flanks and the valleys showed roughness with steeper inclination. After bone insertion, (A) implants showed increased *S*_dq_ values at the tops for all macrogeometries, but only the Strong and Stylus implants were statistically different after placement into bone (*p*=0.035 and *p*=0.004, respectively). Particular attention should be given to the tops of the Strong implant, which showed large increase in the slope of the asperities after bone insertion. It was previously postulated that the contact pressures acting on the tops are higher during bone insertion for this implant due to the reduced top area, and therefore, it is plausible the presence of steeper asperities after bone implantation (Figure 4(b)) and it can be noted that surface finish treatment did not produce a regular topography on the top of those implants. The highest and most dispersed values for *S*_dq_ were observed in the flanks and valleys of the Stylus A and Tryon A implants, and after insertion into the bone, the top of the Stylus and the flank of the Tryon implants were the only regions that showed a significant reduction (*p*=.004 and *p*=.000, respectively).

The present study characterized the surface topography of implants using laser interferometry and scanning electron microscopy before and after insertion into the porcine rib bone. Differences in topography were observed, depending on the area and macrogeometry of the implant. After insertion into bone, surface topography subtly changed in different regions of the screws for all evaluated implants. Within the limitations of this *in vitro* evaluation, it could be observed that the methodology applied herein seemed appropriate for quantitative surface evaluation of different screw-type implants.

### 3.2. Cell Assays

Representative results of the cell proliferation assay are expressed in Table 3, and all showed *p* > 0.05 by the Wilcoxon test.

At 24 h, 48 h, and 96 h, no significant difference was observed between the groups before and after placement of the implants into the porcine bone (*p* > 0.05) for the three types of implants evaluated.

For the cell viability assay, the mean absorbance values (±standard deviation) generated by the test (at 24 h, 48 h, and 96 h) showed no significant difference between groups (B) and (A) for the three types of implants evaluated (*p* > 0.05). Values are presented in Table 4, and all presented *p* > 0.05 on the Wilcoxon test.

Surface treatments, such as double acid etching, may influence cell adhesion, morphology, and proliferation, with a significant increase in osteoblastic cell activity and biocompatibility on porous coatings, favoring the osseointegration process [53, 55]. The surface evaluated in this study had roughness with different characteristics for top, flank, and valley. Nevertheless, cell proliferation and cell viability results showed no significant differences among any of the implant types before and after placement into bone at the time points evaluated.

Osteopontin is a phosphorylated and sulfated glycoprotein, secreted by several cell types including osteoblasts, allowing adhesion of such cells to the extracellular matrix [56, 57]. It is described as an early marker of bone development and osteoblastic differentiation, expressing strongly in the immature matrix, mineralization fronts, having greater expression in osteoblasts [57]. Based on the ELISA assay, the results for osteopontin secretion by the preosteoblastic cells on the different surfaces are shown in Table 5, and all of them presented *p* > 0.05 by the Wilcoxon test.

The results revealed no change in osteopontin expression on any of the surfaces tested (*p* > 0.05). A slight increase in osteopontin at 96 hours could be observed for the three types of implants after installation into the porcine rib, although this did not reach statistical significance. Despite a slight increase in osteopontin at 96 h for all macrogeometries in the implants (B), no difference was significant for the implants (A) at any evaluated time.

As the cell culture method kept the implants in a horizontal position, sedimentation of cells occurred into the valleys and flanks of the threads. Within this study, this factor is regarded as a limitation because it was not possible to accurately assess the behavior of cells at the top, flank, and valley separately, as per the physical surface analysis. In order to assess whether the surface modification caused by insertion into bone alters cell behavior, such methodology was, however, efficient.

Therefore, within the limitations of this study, the results showed herein revealed no significant difference in cell behavior for implant surface before and after the placement of the implant into bone. For future studies, the cell culture method should be optimized to prevent cell sedimentation within the implant threads.

## 4. Conclusions

A subtle change in surface roughness was detected on implants after insertion into bone for all the macrogeometries tested, without significantly affecting the cellular parameters studied.

## Figures and Tables

**Table 1:** Macrogeometric measurements of the implants, where *α*_1_ and *α*_2_ are the internal angles between flanks, and *h* is the thread height.

Implant	Insertion torque (mean ± SD)	Region	*α*_1_ (degrees)	*α*_2_ (degrees)	Height, *h* (*µ*m)	Percentage of area in relation to total implant length
Strong	48.33 N cm ± 10.98	Region 1-cervical	59.3	59.9	331.6	Top: 10.25%
		Region 2-body	59.7	61.7	349.5	Flank: 63.03%
		Region 3-apical	59.7	59.2	250.1	Valley: 26.70%
		*Mean*	**59.9**		**310.4**	
Stylus	33.88 N cm ±15.58	Region 1-cervical	58.7	62.1	389.4	Top: 15.14%
		Region 2-body	59.6	57.1	380.0	Flank: 75.35%
		Region 3-apical	50.7	59.2	283.6	Valley: 9.49%
		*Mean*	**57.9**		**351.0**	
Tryon	33.05 N cm ±13.84	Region 1-cervical	55.8	55.6	350.1	Top: 17.11%
		Region 2-body	55.9	59.7	362.1	Flank: 73.72%
		Region 3-apical	63.2	58.1	312.5	Valley: 9.79%
		*Mean*	**58.0**		**341.6**	

**Table 2:** Surface roughness parameters (*S*_a_*, S*_sk_*, S*_ku_*, S*_tr_, and *S*_dq_) measured in three different areas (top, flank, and valley) in threaded implants before (B) and after (A) insertion into bone using laser interferometry. Mean values ±standard deviation (SD) (^*∗*^*p* < 0.05 between implants before and after insertion).

Implant	Before (B) after (A)	Top	Flank	Valley	Δ total (B-A)
*S*_a_ *µ*m					
Strong	B	0.21 ± 0.02	0.69 ± 0.11	0.57 ± 0.24	0.02
	A	0.55 ± 1.21	0.75 ± 0.31	1.35 ± 1.55^*∗*^	
Stylus	B	0.23 ± 0.03	0.70 ± 0.10	0.26 ± 0.13	0.42
	A	0.16 ± 0.11^*∗*^	0.99 ± 1.04	2.38 ± 3.85	
Tryon	B	0.48 ± 0.26	1.98 ± 0.68	0.10 ± 0.07	0.17
	A	0.47 ± 0.64	1.25 ± 0.46^*∗*^	3.68 ± 2.46^*∗*^	
*S*_sk_					
Strong	B	−0.34 ± 0.31	0.06 ± 0.37	−0.12 ± 0.27	−0.01
	A	−0.20 ± 0.39	0.01 ± 0.31	−0.14 ± 0.38	
Stylus	B	−0.17 ± 0.19	0.07 ± 0.51	−0.13 ± 0.52	0.01
	A	−0.17 ± 0.20	0.06 ± 0.61	−0.63 ± 1.48	
Tryon	B	−0.12 ± 0.45	0.06 ± 0.57	−0.02 ± 0.32	−0.02
	A	−0.11 ± 0.61	0.19 ± 0.63	0.17 ± 0.82	
*S*_ku_					
Strong	B	4.21 ± 1.45	3.23 ± 0.81	3.58 ± 0.49	−0.04
	A	13.54 ± 4.88	3.50 ± 0.73	3.40 ± 0.99	
Stylus	B	3.63 ± 0.50	3.36 ± 4.60	3.52 ± 1.06	−0.02
	A	6.99 ± 4.36^*∗*^	9.54 ± 6.68^*∗*^	17.64 ± 33.52^*∗*^	
Tryon	B	4.60 ± 1.75	3.69 ± 1.78	3.01 ± 0.56	−0.05
	A	8.48 ± 7.18^*∗*^	4.72 ± 3.21	4.92 ± 5.89	
*S*_tr_ *µ*m					
Strong	B	0.21 ± 0.05	0.10 ± 0,13	0.17 ± 0.07	0.01
	A	0.22 ± 0.14	0.05 ± 0,06	0.19 ± 0.18	
Stylus	B	0.21 ± 0.05	0.08 ± 0.01	0.09 ± 0.03	0.01
	A	0.21 ± 0.11	0.03 ± 0.02^*∗*^	0.03 ± 0.02^*∗*^	
Tryon	B	0.15 ± 0.10	0.11 ± 0.11	0.08 ± 0.02	−0.01
	A	0.15 ± 0.10	0.04 ± 0.01^*∗*^	0.04 ± 0.02^*∗*^	
*S*_dq_ *µ*m					
Strong	B	0.03 ± 0.03	1.12 ± 0.19	0.40 ± 0.08	−0.05
	A	0.53 ± 0.34^*∗*^	1.20 ± 0.58	2.07 ± 2.30^*∗*^	
Stylus	B	0.42 ± 0.05	1.14 ± 1.11	5.02 ± 2.27	0.53
	A	0.36 ± 0.11^*∗*^	1.80 ± 1.66^*∗*^	4.67 ± 7.14	
Tryon	B	0.85 ± 0.46	3.15 ± 1.11	1.15 ± 0.40	−0.01
	A	0.88 ± 0.99	1.95 ± 0.73^*∗*^	5.77 ± 4.40^*∗*^	

**Table 3:** Mean (±standard deviation) of cell proliferation on implants B and A by the trypan blue vital exclusion method in osteoblasts.

Time	Cell × 10^4^ mean ± SD	Cell × 10^4^ mean ± SD
	*Strong B*	*Strong A*
24 h	1.25 ± 0.46	1.77 ± 0.45
48 h	5.25 ± 0.34	4.07 ± 0.26
96 h	8.22 ± 0.22	8.14 ± 0.56
	*Stylus B*	*Stylus A*
24 h	2.14 ± 0.46	1.48 ± 0.25
48 h	5.62 ± 0.25	4.22 ± 0.22
96 h	8.81 ± 0.46	9.03 ± 0.26
	*Tryon B*	*Tryon A*
24 h	2.07 ± 0.13	1.63 ± 0.34
48 h	6.00 ± 0.23	3.55 ± 0.22
96 h	8.88 ± 0.67	9.40 ± 0.34

**Table 4:** Mean (±standard deviation) of cell viability using the MTT assay in osteoblasts.

Time	Optical density = 590 nm mean ± SD	Optical density = 590 nm mean ± SD
	*Strong A*	*Strong D*
24 h	0.16 ± 0.00	0.11 ± 0.01
48 h	0.40 ± 0.03	0.30 ± 0.03
96 h	0.82 ± 0.02	0.73 ± 0.06
	*Stylus A*	*Stylus D*
24 h	0.15 ± 0.01	0.12 ± 0.02
48 h	0.39 ± 0.03	0.29 ± 0.04
96 h	1.00 ± 0.00	0.58 ± 0.09
	*Tryon A*	*Tryon D*
24 h	0.16 ± 0.02	0.11 ± 0.00
48 h	0.39 ± 0.01	0.30 ± 0.03
96 h	0.87 ± 0.01	0.73 ± 0.13

**Table 5:** Mean (±standard deviation) in pg/mL of osteopontin synthesis on implants B and A as quantified via the ELISA assay.

Time	Mean ± SD	Mean ± SD
	*Strong B*	*Strong A*
24 h	986.28 ± 81.42	1103.2 ± 34.21
48 h	156.75 ± 27.99	1321.12 ± 117.46
96 h	1224.72 ± 161.74	1941.33 ± 107.66
	*Stylus B*	*Stylus A*
24 h	1120.01 ± 54.63	1222.85 ± 15.38
48 h	1891.62 ± 54.72	1266.86 ± 104.97
96 h	1292.05 ± 59.8	1935.2 ± 116.28
	*Tryon B*	*Tryon A*
24 h	964.15 ± 220.98	1171.05 ± 96.22
48 h	1769.25 ± 146.94	1390.77 ± 159.43
96 h	1360.34 ± 58.66	2078.06 ± 131.64

## Data Availability

The data used to support the findings of this study are included within the article.
